# Direct Ink Writing 3D Printing Polytetrafluoroethylene/Polydimethylsiloxane Membrane with Anisotropic Surface Wettability and Its Application in Oil–Water Separation

**DOI:** 10.3390/polym17020174

**Published:** 2025-01-13

**Authors:** Peng Geng, Chengjian Jiang

**Affiliations:** State Key Laboratory of Material Processing and Die & Mold Technology, School of Materials Science and Engineering, Huazhong University of Science and Technology, Wuhan 430074, China; jiang_cj@hust.edu.cn

**Keywords:** DIW printing, PTFE/PDMS, anisotropic surface wettability, oil–water separation

## Abstract

Biological surfaces with physical discontinuity or chemical heterogeneity possess special wettability in the form of anisotropic wetting behavior. However, there are several challenges in designing and manufacturing samples with anisotropic wettability. This study investigates the fabrication of PTFE/PDMS grid membranes using Direct Ink Writing (DIW) 3D printing for oil–water separation applications. The ink’s rheological properties were optimized, revealing that a 60% PTFE/PDMS composite exhibited the ideal shear-thinning behavior for 3D printing. Our research investigated the interplay between various printing parameters like the extrusion air pressure, layer thickness, feed rate, and printing speed, which were found to influence the filament dimensions, pore sizes, and hydrophobic properties of the grid membrane. Two distinct grid structures were analyzed for their wettability and anisotropic hydrophobic characteristics. The grid membranes achieved up to 100% oil–water separation efficiency in specific configurations. Separation efficiency was shown to be dependent on factors like intrusion pressure, grid architecture, and the number of layers. This study underscores the potential of DIW 3D printing in creating specialized surfaces with controlled wettability, particularly superhydrophobicity and anisotropy, paving the way for advanced environmental applications such as efficient oil–water separation.

## 1. Introduction

Wettability is a pivotal characteristic of solid surfaces, governed by their chemical composition and geometric microstructures [1,2]. Surface chemistry dictates that wettability hinges on the configuration of atoms or atomic groups on the surface’s outermost layer [3]. Typically, non-polar polymers, due to their inherent attributes, manifest hydrophobic or superhydrophobic tendencies. Beyond conventional hydrophobic and hydrophilic behaviors, researchers have focused on investigating unique surface wettability types, such as anisotropic and controllable wettability [4,5,6]. Anisotropic wettability results in variable contact angles in different directions, leading to directional droplet diffusion or movement. Numerous natural organisms demonstrate this particular wetting phenomenon due to their surface’s physical discontinuity or chemical heterogeneity. For instance, water droplets on rice leaves [7] and butterfly wings [8] move in a designated direction, and spider silk’s intricate structures [9] or the nepenthes’ peristome surface [10] direct water droplets consistently. Such distinctive wettability has profound applications in directional liquid transportation, waterproofing, and microfluidic technologies [11,12,13].

Drawing inspiration from the anisotropic wettability inherent in biological surfaces, considerable advancements have been realized by researchers in devising and fabricating micro- and nanostructures exhibiting optimal anisotropic wettability. To achieve superhydrophobic surfaces, for instance, low-surface-energy materials have been subjected to treatments such as chemical etching, laser processing, and spray coating to generate roughened surfaces endowed with micro- and nanostructures [14,15]. In the quest to fabricate anisotropic wetting surfaces, researchers have employed techniques such as electrospinning [16], laser etching [17], and soft lithography [18] to create asymmetric geometric structures. However, such methodologies often carry hefty price tags and present challenges, including multi-stage processing, restrictions in the processing area, and inconsistent structural control. Conversely, the ascendance of 3D-printing technology in recent years, notably DIW 3D printing [19,20], offers a host of benefits, from cost-effectiveness and heightened efficiency to diverse materials. Through meticulous tweaking of the printing software and operational parameters, it is feasible to achieve structures with bespoke surface wettability features [21,22,23,24,25]. Building upon these advancements, the selection of materials for 3D printing plays a crucial role in determining the final properties of the printed structures.

The superhydrophobic properties of a surface are not only dictated by its micro- and nanostructures but are also significantly influenced by the material’s inherent attributes. PDMS is extensively utilized across various research fields owing to its desirable attributes, including easy fabrication, high flexibility, thermal stability, and inherent hydrophobicity [26,27,28]. These characteristics make PDMS an ideal matrix phase for DIW 3D printing, enabling the realization of a multitude of functionalities upon compounding with other materials. PTFE, known for its exceptional thermal and chemical stability as well as its inherent superhydrophobic properties, has been effectively employed in the fabrication processes for desalination applications [29,30]. However, the processing of PTFE presents certain challenges. Consequently, the combination of PDMS and PTFE emerges as a promising candidate for the design of porous superhydrophobic membranes, which are highly sought after for their efficacy in oil–water separation applications.

To address this approach’s limitations, several researchers have tried to apply DIW 3D printing in the fabrication of oil–water separation structures. Ma et al. utilized DIW 3D printing to create flexible surfaces inspired by shark skin, enhancing drag reduction and antifouling capabilities with modified PDMS ink [31]. Shin et al. employed 3D printing and sacrificial molding to fabricate a bio-inspired PDMS sponge with exceptional hydrophobicity and oleophilicity [32]. Huang et al. developed superhydrophobic elastomeric meshes through DIW 3D printing, showcasing tunable water repellency and anisotropic wettability, which maintained hydrophobicity under deformation [33]. While these studies highlight the potential of DIW 3D printing in oil–water separation, they have not extensively analyzed the dynamic hydrophobic characteristics of the separation grids or provided a comprehensive understanding of the underlying separation performance and mechanisms.

In this study, we prepared a thixotropic polytetrafluoroethylene/polydimethylsiloxane (PTFE/PDMS) composite ink by combining the non-polar polymer PTFE with PDMS polymers. Utilizing this PTFE/PDMS ink, a grid membrane exhibiting unique surface wettability traits was fabricated through DIW 3D printing. When we modified the printing parameters to regulate the filament width and pore size, the printed grid membranes, when subjected to varying parameters, exhibited distinct water contact angles (WCAs) in both parallel and vertical orientations. Notably, this led to the manifestation of anisotropic surface wetting properties. The 3D-printed PTFE/PDMS grid membrane was subsequently applied with great efficacy in an oil–water separation application.

## 2. Materials and Methods

### 2.1. Preparation of the PTFE/PDMS Ink

PDMS (Sylgard 184) was sourced from Dow Corning Corporation (Midland, MI, USA), while the remaining chemicals were acquired from Macklin Biochemical Technology Co., Ltd. (Shanghai, China). The PDMS prepolymer (Part A), curing agent (Part B), and inhibitor (3-butyn-1-ol, 97%) were combined in a ratio of 100:10:1. Subsequently, a specified amount of PTFE micro-powder (with an average particle size of <1 μm) was incrementally added. The blend was mechanically agitated at 650 r/min for 30 min to achieve a uniformly mixed PTFE/PDMS ink. Once prepared, the ink was transferred to a syringe barrel and centrifuged using a centrifugal planetary mixer at 5000 r/min for 3 min to eliminate residual bubbles. The inhibitor served to neutralize the platinum catalyst in the PDMS, extending the ink’s operational duration. All chemicals were utilized as received without additional purification.

### 2.2. DIW 3D Printing of PTFE/PDMS Grid Membranes

**Figure 1:**
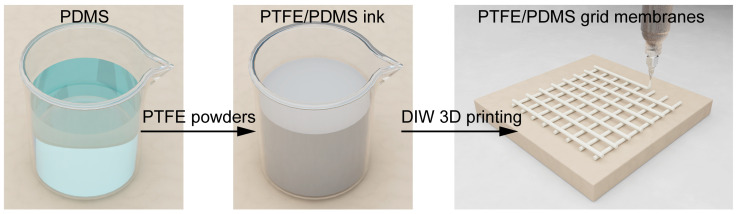
Preparation and DIW 3D printing of PTFE/PDMS composite inks.

The self-built DIW 3D printer comprised an extrusion module (which included a syringe barrel, the printing ink, and a dispensing machine needle with an inner diameter of 250 μm), a three-dimensional motion platform, a pneumatic extrusion system, and integrated software control modules. As per the pre-set path, the print base maneuvered along the X- and Y-axes, while with each subsequent layer, the needle ascended along the Z-axis. Concurrently, through regulated air pressure, the needle dispensed the PTFE/PDMS ink, ensuring the accurate placement of inks with specific rheological attributes onto the print base, thus generating the desired patterns. Upon printing, the sample was subjected to a heat-curing process at 120 °C for 30 min and was then carefully removed from the platform. Figure 1 depicts the preparation and printing procedures of the PTFE/PDMS composite materials.

To elucidate the influence of filament topology on sample attributes such as porosity and hydrophobicity, two distinct infill patterns (Structure 1 and Structure 2, respectively) were printed (Figure S1, Supporting Information). To safeguard the integrity of un-cured material during printing, the printing path was optimized to avoid intra-layer filament intersections. Structure 1 requires a minimum of two layers with filaments oriented perpendicularly to each other to constitute the fundamental functional unit, while Structure 2 is capable of forming a functional unit with a single layer. To facilitate the comparison of their performances, both structures were printed with at least two layers for characterization.

### 2.3. Characterization

For morphology characterization via scanning electron microscopy (SEM), a Nova NanoSEM 450 (FEI Company, Hillsboro, OR, USA) instrument, equipped with an energy-dispersive X-ray spectrometer, was utilized to examine both the surface and cross-sectional morphologies of the printed grid membranes.

Rheological properties of the PTFE/PDMS ink were assessed using a HAAKE MARS40 rotary rheometer (Thermo Fisher Scientific Inc., Waltham, MA, USA). The relationship between apparent viscosity and shear rate was evaluated in the control rate mode for a duration of 12 min. Meanwhile, the correlation between the storage modulus (G′) and the loss modulus (G′′) and shear stress was gauged in the control stress mode over 6 min at an oscillation frequency of 1 Hz.

Fourier transform infrared (FTIR) spectroscopy measurements were obtained with a Nicolet 6700 spectrometer (Thermo Fisher Scientific, Waltham, MA, USA). The FTIR spectra for the PTFE micro-powders were recorded in the 4000–400 cm^−1^ range using KBr compression, while the PTFE/PDMS composites were characterized within the 4000–600 cm^−1^ range.

Differential scanning calorimetry (DSC) measurements were conducted on PDMS-based inks using a DSC25 differential scanning calorimeter (TA Instruments, New Castle, DE, USA). The non-isothermal curing test proceeded at a rate of 10 °C/min from room temperature (RT) to 200 °C. For the isothermal curing test, the temperature was rapidly raised to 120 °C within a minute and then sustained for 10 min. By evaluating the thermal curing behavior of the inks, the curing temperature and duration for the PDMS-based inks with varying PTFE contents were determined.

Tensile tests were conducted at a rate of 5 mm/min following EN ISO 527 using an E1000 electric dynamic test instrument (Instron, Norwood, MA, USA). The PTFE/PDMS ink, containing varying PTFE contents, was vacuumed and centrifuged to eliminate air bubbles. Subsequently, the ink was poured into a mold to fabricate dog-bone-shaped tensile testing samples.

For contact angle characterization, the WCAs on sample surfaces were measured using a DSA100 contact angle measuring instrument (KRUSS GmbH, Hamburg, Germany) at RT. Measurements were taken at three distinct points on the sample to calculate average values. For contact angle characterization under varying strain conditions, the PTFE/PDMS grid membrane was securely positioned horizontally between two tensile clamps. The distance between the clamps represented the unstrained grid membrane length, and the associated contact angle was measured. Upon stretching one clamp end, both the grid membrane length and CA were measured.

## 3. Results and Discussion

### 3.1. Printability of the PTFE/PDMS Ink

The PDMS prepolymer, curing agent, and inhibitor were combined, followed by the addition of different contents of PTFE powder. This inclusion was intended to both ensure the ink’s pronounced shear-thinning behavior and enhance the hydrophobic properties of the 3D-printed sample. The final composite ink underwent processing via a DIW 3D-printing device (Figure S2, Supporting Information).

**Figure 2:**
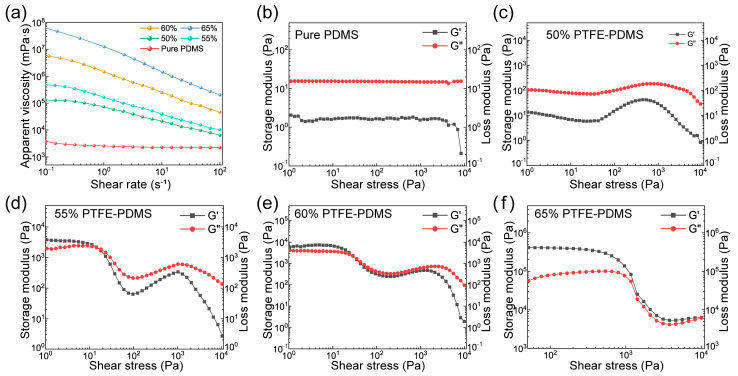
Rheological properties of the PTFE/PDMS composite ink. (**a**) Relationship between apparent viscosity and shear rate for PDMS-based composite inks with different PTFE contents; (**b**–**f**) the storage modulus (G′) and loss modulus (G′′) of the PDMS-based inks with different PTFE contents (0%, 50%, 55%, 60%, and 65%) as a function of shear stress.

Figure 2 illustrates the rheological properties of PDMS-based inks with varied PTFE contents. The ink’s viscosity shifted logarithmically with the shear rate. For varying shear rates, pure PDMS ink’s viscosity consistently ranged between 2000 and 3000 mPa·s, showing no distinct correlation with the shear rate (as shown in Figure 2a). When incorporating PTFE into the ink, samples containing 50–65% PTFE/PDMS demonstrated shear-thinning characteristics. Concurrently, the viscosity of the ink was augmented in line with the PTFE concentration. Given the comparable surface tensions of PDMS (19.67 mN/m at 20 °C) [34] and PTFE (23.18 mN/m at 20 °C) [35], a relatively stable contact interface layer naturally formed [36]. This allowed for the uniform dispersion of PTFE micro-powders in PDMS, ensuring no phase separation. During the DIW 3D-printing process, PDMS-based inks only with specific PTFE contents showcased commendable printing compatibility, facilitating the steady extrusion of filament for a stable grid membrane structure. Nevertheless, excessive PTFE powder inclusion resulted in heightened ink viscosity, impeding its passage through the micro-needle and causing extrusion failures. As such, examining the impact of the PTFE concentration on the ink’s rheological properties becomes imperative.

The rheological properties of the DIW 3D-printing inks such as the shear thinning, viscoelastic inversion, and flow yield stress have shown to be the main factors that affect the stability of 3D-printed samples [37,38]. The rheological properties of shear thinning can be described by the Hershel–Bulkley model and expressed by the following equation:

(1) $$\begin{array}{cc} \overset{˙}{r}=0 & \;\mathrm{if}\;\tau <\tau_{y}\\ \tau =\tau_{y}+K{\overset{˙}{r}}^{n} & \;\mathrm{if}\;\tau \geq \tau_{y} \end{array}$$

The Herschel–Bulkley model describes *η*, the apparent viscosity of the fluid, as follows:

(2) $$\eta =\frac{\tau_{y}}{\dot{r}}+K{\dot{r}}^{n-1},$$

where *τ_y_* is the yield stress, *K* is the consistency coefficient, $\dot{r}$ is the shear rate, *n* is the flow behavior index, and *η* is the apparent viscosity. When *n* < 1, the viscosity decreased with an increased shear rate, satisfying the property of shear thinning. This property enabled the ink in the needle to maintain a low viscosity under shear stress, so that it could be smoothly extruded. After extrusion, the shear stress was removed and the viscosity of the filament increased, resulting in a preserved shape and stabilized structure.

However, not all inks with shear-thinning properties demonstrate optimal printing adaptability. Critical to this adaptability is the relationship between the storage modulus (G′) and the loss modulus (G′′) during the extrusion process. The G′ of pure PDMS ink and 50% PTFE/PDMS consistently remains below the G′′. This suggests that the ink retains a viscous, liquid-like state across a spectrum of shear stresses. While this allows for easy extrusion from the needle, the absence of thixotropic properties impedes the ink’s ability to maintain the 3D-printed structure’s integrity. Conversely, inks with high PTFE contents like 55% PTFE/PDMS, 60% PTFE/PDMS, and 65% PTFE/PDMS (as can be seen in Figure 2d–f) exhibit a G′ that exceeds the G′′ under low-shear-stress conditions. This imparts solid-like attributes to the ink, beneficial for shape retention post-printing [39,40]. With escalating shear stress, however, this relationship inverts, leading to a more liquid-like flow of the ink—a property beneficial for extrusion through the printing nozzle. For example, at shear stress levels (<~300 Pa), the 55% PTFE/PDMS ink exhibits a liquid state. However, once the shear stress exceeds 300 Pa, there is an evident viscoelastic inversion, leading the ink to resemble a solid-like state. This makes it amenable to printing. The 60% PTFE/PDMS ink mirrors the rheological characteristics of the 55% ink, transitioning from solid to liquid as stress increases. The transition point for the 60% PTFE/PDMS is approximately 30 Pa. The 65% PTFE/PDMS, with a transition point at a much higher 10,000 Pa, primarily behaves as a solid. Its stability, while commendable, complicates its extrusion through the printing nozzle, rendering it suboptimal for DIW 3D printing. Here, rheological investigations identified 60% PTFE/PDMS inks as best suited to DIW 3D printing, given their smooth extrusion and shape retention.

### 3.2. Material Characterization

**Figure 3:**
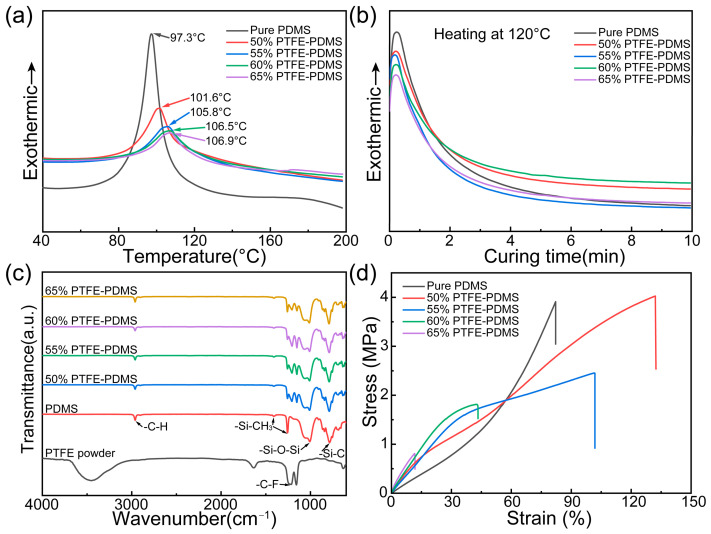
Material characterization of PTFE/PDMS composite inks. (**a**) Non-isothermal and (**b**) isothermal DSC curves for pure PDMS and the PTFE/PDMS composite; (**c**) FTIR spectra of the PTFE powder and PTFE/PDMS grid membrane with different PTFE contents; (**d**) stress–strain curves of PTFE/PDMS samples with different PTFE contents.

The curing performance of the ink is crucial, necessitating the determination of the optimal curing temperature and time through DSC non-isothermal and isothermal curing tests (Figure 3a,b). The curing for pure PDMS initiates around 60 °C, peaking in reaction rate at an exothermic peak temperature of 97.3 °C. As the PTFE content increases, there is a corresponding shift in the ink’s heat release peak to higher temperatures: 101.6 °C at 50% PTFE, 105.8 °C at 55% PTFE, 106.5 °C at 60% PTFE, and 106.9 °C at 65% PTFE. These findings suggest that PTFE powder addition marginally inhibits the PDMS ink’s curing reaction. Compared to the 97.3 °C curing temperature of pure PDMS, the influence of the PTFE content on the curing temperature remains relatively minimal, with PDMS crosslinking persisting as the primary reaction. Finally, we chose 120 °C as the heating temperature to ensure rapid curing of the ink. The isothermal DSC curves for the PDMS based inks reveal that, at 120 °C, all inks achieved their peak curing rate within a minute and predominantly finalized within 6 min. Consequently, a curing condition of 120 °C for 10 min ensures thorough curing of the PDMS-based inks.

Figure 3c presents the FTIR spectra of the PTFE powder and the printing grid membranes across varied PTFE contents. Infrared absorption peaks near the wave-number regions of 1218 and 1155 cm^−1^ were due to the antisymmetric and symmetric stretching vibrations of the CF_2_ group, respectively. The absorption peak proximate to 1008 cm^−1^ in the 3D-printed grid membrane can be attributed to the tensile vibrations of Si-O-Si. Moreover, the peaks at around 1257 and 1412 cm^−1^ result from deformation and asymmetric deformation vibrations of the two CH_3_ groups linked to Si. The distinct peaks near 786 and 2962 cm^−1^ signify the tensile vibrations of Si-C and C-H, respectively. Compared with the spectra of pure PDMS, the characteristic infrared absorption of the PTFE/PDMS composites (near 1215 and 1157 cm^−1^) intensifies as the PTFE content rises. Furthermore, in contrasting the spectra of PTFE and PDMS, no emergent infrared absorption peaks were observed for the PTFE/PDMS composites, implying that PTFE was merely physically incorporated into the polymer matrix.

Figure 3d and Table 1 shows the stress–strain curves for PTFE/PDMS materials with different PTFE contents. All samples demonstrate commendable elongation characteristics. The fracture elongation of pure PDMS reached 82%. However, introducing PTFE powder reduced the fracture elongation of PTFE/PDMS samples while enhancing the tensile strength. At a 50% PTFE content, PTFE/PDMS exhibited superior tensile properties, registering a tensile strength of 4.0 MPa and a fracture elongation of 132%. A rise in PTFE content correlated with diminishing tensile strength and fracture elongation of PTFE/PDMS samples. The increased presence of heterogeneous material interfaces in the PTFE/PDMS samples due to a heightened PTFE concentration is responsible for the decline in tensile strength. Another factor to consider is the heightened viscosity in PTFE/PDMS samples with a higher PTFE content, which can introduce hole defects during casting. In contrast to pure PDMS, PTFE/PDMS materials exhibited higher tensile strength, because of the compatibility of physical properties between PTFE and PDMS. The aggregation morphology of PTFE particles, their dispersibility within the matrix polymer, and the interfacial compatibility are pivotal factors influencing the physical properties of composite membranes. SEM analysis revealed that the PTFE-filled PDMS polymers exhibited a favorable dispersion and interfacial compatibility. Consequently, at a PTFE content of 50 wt%, there was a notable enhancement in both tensile stress and elongation of the composite membrane. However, when the filler content exceeds an optimal level, the interconnection of PTFE particles can disrupt the continuity of the PDMS phase within the membrane, leading to a degradation of its properties [41].

**Figure 4:**
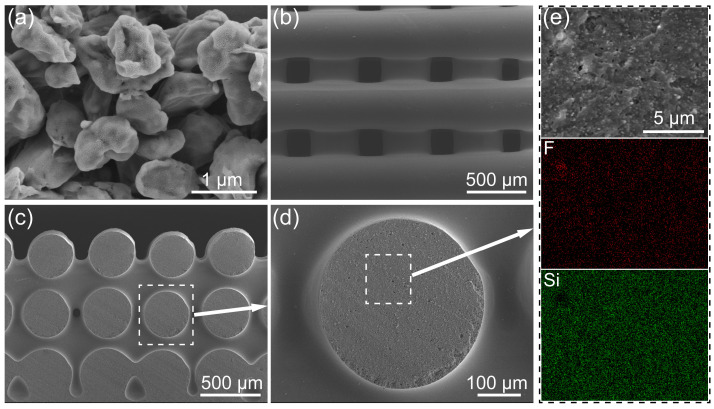
SEM images. (**a**) PTFE powder morphology; (**b**–**e**) microstructural characteristics of DIW 3D-printed PTFE/PDMS grid membranes.

Figure 4 shows the microstructure of the PTFE powder and the 3D-printed PTFE/PDMS sample. The PTFE micro-powder comprises irregular particle clusters with an average diameter below 1 μm. Figure 4b–e show the 3D-printed sample with a distinct grid pattern, emphasizing excellent adhesion between filaments across neighboring layers. This filament adhesion not only enhances the surface finish but also augments the sample’s mechanical strength. Figure 4c offers insight into the three-layer grid membrane from a fractured section. An enlarged view of the filament surface indicates the PTFE micro-powder uniformly dispersed within the PDMS matrix. The SEM image combined with the EDS elemental mapping of a single filament cross-section (Figure 4d,e) confirms the homogeneous dispersion of PTFE powder within the PDMS matrix. Such a uniform distribution underscores the intimate interaction and compatibility between PTFE and PDMS, pivotal in defect minimization and mechanical reinforcement of the composites.

### 3.3. DIW 3D Printing of the PTFE/PDMS Grid Membrane

To ascertain the optimal DIW 3D-printing parameters for PTFE/PDMS, we undertook a systematic investigation of the extrusion process, divided into four sequential steps. Firstly, a PTFE/PDMS filament was extruded onto a water surface under varied extrusion pressures (*P*) from 550 to 800 kPa. This method was adopted to minimize the influence of gravitational forces on the length of the extruded filament. Evaluating the length of the extruded filament allowed us to calculate the feed rate (*F*) for different air pressures. Secondly, the filament was extruded by adjusting the ratio (*R*) between the printing speed (*v*) and the feed rate (*F*), covering a range of 0.5, 0.75, 1.0, 1.25, and 1.5. This approach primarily concentrated on individual filament extrusion. Then, the effect of printing parameters on the grid membrane was evaluated. Thirdly, parallel filaments of diverse layer thicknesses (*LT*: 0.15 mm, 0.25 mm, 0.35 mm) and filament distances (*FD*) (between 300 and 1000 μm) were printed. After heat treatment, the spacing between the filaments (*FS*) was measured. It is essential to note that FS signifies the gap between successive parallel filaments. Thus, the filament width (*FW*) is derived as *FD* minus *FS*. Using the optimal printing parameters, a grid membrane of assorted fill patterns and numbers of layers was fabricated.

**Figure 5:**
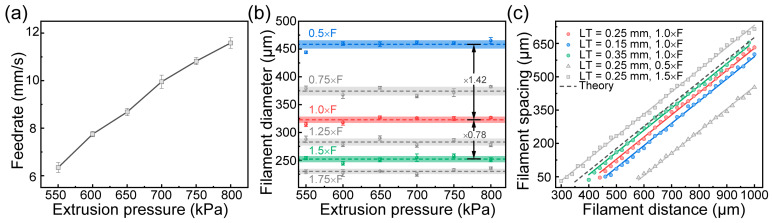
DIW 3D-printing parameters of the PTFE/PDMS grid membrane. (**a**) Influence of extrusion air pressure on filament diameter; regulation of (**b**) filament diameter and (**c**) filament spacing.

PTFE/PDMS ink with a 60% content was extruded onto a water surface (Figure S3, Supporting Information). The *F* for various extrusion air pressure conditions was determined by measuring the length of the extruded filament. The findings revealed a significant positive linear correlation between *F* and *P* (as shown in Figure 5a). This is consistent with the phenomenon of filament swelling accompanied by the increased extrusion air pressure during the extrusion process in traditional DIW printing.

In DIW 3D-printing strategies, ensuring that *F* aligns with *v* is imperative. If *v* ≤ *F,* filament coiling and accumulation can occur. On the other hand, a feed rate below the printing speed might cause the filament to thin out or become discontinuous [42]. To facilitate uniform and precise filament extrusion, the diameter of a PTFE/PDMS filament was assessed at varying printing speeds, as shown in Figure 5b. The findings indicate that when *v* = *F*, the filament diameter (322.90 ± 5.71 μm) remains consistent, irrespective of extrusion pressure variations. Additionally, adjusting *R* can also modify the filament diameter. For instance, when *v* = 1.5 × *F* (*R* = 1.5), the filament diameter decreases to 252.15 ± 5.61 μm, which is approximately 0.78 times the optimal diameter. When *v* = 0.5 × *F* (*R* = 0.5), the filament diameter increases to 458.33 ± 7.52 μm, which is approximately 1.42 times the optimal diameter.

When using PTFE/PDMS ink with a specific powder content, the diameter of the extruded filament can be regulated by altering *R*, avoiding the need for extrusion air pressure adjustments. Traditional pneumatic extrusion DIW is frequently considered challenging and imprecise when using air pressure changes to control filament diameter. However, modifying *R*, as demonstrated by the prior results, is both straightforward and precise.

The filament width (*FW*) and filament spacing (*FS*) influence the topological structure of the grid membrane. Adjusting the pore size of the grid membrane through the filament distance (*FD*) offers a practical method to control the membrane’s surface wettability. Ideally, the pore size equates to *FD* minus *FW*. Nevertheless, the actual pore size during the printing process may differ from theoretical values due to factors like filament adhesion, diffusion, and collapse. To assess the impact of process parameters on *FW*, a PTFE/PDMS ink with a 60% content was 3D-printed under varied layer thicknesses (*LT*) and printing speeds (*v*), employing 650 kPa of air pressure. Figure 5c depicts the relationship between *FD* and *FS* (also shown in Figure S4, Supporting Information). The point where the fitted line intersects the X-axis indicates the *FW* for specific printing conditions. Notably, *FS* amplifies with increased *R* or *LT*. The *FS* is governed by a synergistic interplay of factors, including filament stretching, accumulation, and the compression effect of the nozzle during extrusion (Figure S5, Supporting Information). When considering *LT* = 0.35 mm and *R* = 1, the *FS* (represented by the green line) is consistent with theoretical values after eliminating the influence of nozzle compression. For *LT* values such as 0.15 mm or 0.25 mm, the nozzle compression effect becomes non-negligible. Data from the fitted curves suggest *FW* values of 409.60 μm (*LT* = 0.15 mm), 371.43 μm (*LT* = 0.25 mm), and 337.01 μm (*LT* = 0.35 mm). With a constant *LT*, changing *R* can alter *FW*. Specifically, at *LT* = 0.25 mm and *R* = 1, adjusting to *R* = 1.5 results in an *FW* of 267.97 μm, due to the combined influence of stretching and nozzle compression.

Based on the aforementioned analysis, control over both the filament width and spacing can be achieved through adjustments in layer thickness and the ratio between the printing speed and feed rate, analogous to the methodology employed for regulating the dimensions of extruded filaments.

**Figure 6:**
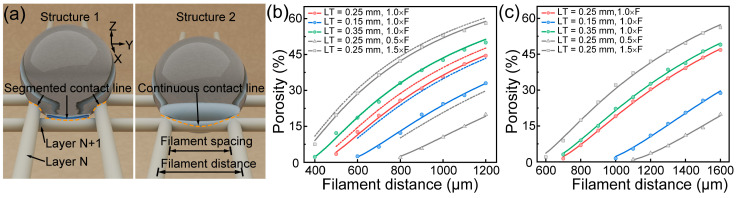
DIW 3D printing of the PTFE/PDMS grid membrane with different infill patterns. (**a**) Schematic illustration of grid membranes of different infill patterns; porosity of the (**b**) Structure 1 and (**c**) Structure 2 PTFE/PDMS grid membranes.

The filament serves as the foundational unit for creating pores and grids, with the topological interrelationships among filaments playing critical roles in determining a sample’s hydrophobic properties and oil–water separation efficiency. For grid membranes of varying patterns (as shown in Figure 6a), porosity serves as an indicator of their structural characteristics. The correlation between *FS* and porosity was investigated, as shown in Figure 6b,c. The dashed lines in the figure represent theoretical porosities for the two grid structures. It was observed that when *LT* = 0.35 mm and *R* = 1, the measured porosity of Structure 1 aligned closely with the theoretical value. Deviations occurred with alterations in layer thickness or printing speed, consistent with the mechanism of altered filament spacing in Figure 5b. These deviations are attributable to the interplay of nozzle compression, filament tension, and material accumulation. Therefore, adjusting *LT* and *R* offers a means of adjusting porosity. Compared to Structure 1, Structure 2 exhibited lower porosity under an equivalent *FS*, consequently diminishing the effective area for oil–water separation. This reduction is caused by the high-speed alternating motion of the printing nozzle in the X and Y directions, resulting in deformation of intra-layer pores and misalignment of inter-layer pores.

In the DIW printing process, nozzle compression during movement in the X/Y direction can induce variations in filament width but also enhances interlayer bonding in the 3D-printed sample. Although a greater layer thickness facilitates precise control over filament width, it introduces the risk of cumulative deviations, potentially leading to structural instability as the number of layers grows. Accordingly, based on preceding studies on filament and grid structures, *LT* = 0.25 mm and *R* = 1 were selected for hydrophobicity and oil–water separation experiments.

### 3.4. Wetting Behavior of the PTFE/PDMS Grid Membrane

**Figure 7:**
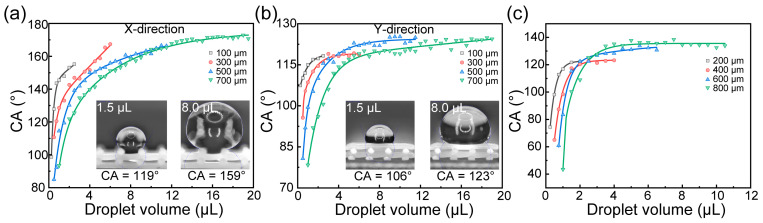
CA measurements for PTFE/PDMS grid membranes. CA of the grid membrane of Structure 1 in the (**a**) X direction and (**b**) Y direction; (**c**) CA of the PTFE/PDMS grid membrane of Structure 2.

The CA of a sample is influenced by both the inherent wettability of the material and the surface’s microstructure. During contact angle measurements, droplet behavior is subject to surface tension and gravitational forces. The effect of gravity becomes negligible for small droplets (≤2 μL) but significant for larger volumes (≥5 μL). In DIW 3D-printed PTFE/PDMS grid membranes, the gas phase is trapped in the cavities between intersecting filaments, cooperating with the solid phase to support the liquid, resulting in a composite Cassie–Baxter (CB) wetting state. The liquid–gas interface above the cavity adheres to the solid phase and deforms under pressure, a phenomenon commonly referred to as meniscus. This state is metastable and can transition to the Wenzel wetting state, resulting in the sessile droplet depinning and the cavity filling with liquid [43]. Unlike traditional microstructured surfaces, the DIW 3D-printed grid membranes have larger dimensions and their porosity is influenced by printing parameters. The relationship between contact angles, filament spacing, droplet sizes, and infill patterns was examined (as shown in Figure 7 and Figure S6, Supporting Information).

During CA measurements, droplets remain consistently positioned between adjacent filaments. Structure 1 exhibits greater CAs in the X direction compared to the Y direction, a feature absent from Structure 2. A rapid increase in the CA is observed as the droplet volume increases, plateauing beyond a specific threshold. For instance, in Structure 1 with an *FS* of 500 μm, the CA is 85.1° at a droplet volume of 0.25 μL and ascends to 119.4° at 1.5 μL. Beyond this point, the CA rises slowly, reaching a maximum of 166.4° at a droplet volume of 11.5 μL. Initially, the droplet is pinned between adjacent filaments and adopts a flattened shape due to surface tension. As the liquid volume increases, the three-phase contact line shifts along the filament’s contour, resulting in an ellipsoidal droplet. Further increases in liquid volume led to gravitational compression of the droplet, thereby augmenting the CA. In contrast, the CA in the Y direction rises only from 80.8° to 125.0°, and the angle disparity between the two directions broadens from 5.1° to 41.4°. Structure 2 grids display consistent variations in CA with the droplet volume, similar to Structure 1.

**Figure 8:**
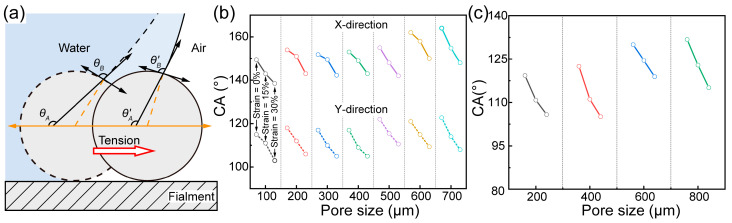
CA measurements for the PTFE/PDMS grid membrane under strain conditions. (**a**) Schematic illustration of the variation in CA and the grid membrane; CA of PTFE/PDMS grid membranes of (**b**) Structure 1 and (**c**) Structure 2.

Figure 8 illustrates CAs under varying strain conditions. With the liquid volume maintained at 60% of its maximum droplet volume that the grid membrane can support, CA decreases with increasing grid membrane strain, ranging from 0% to 30% (as shown in Figure S7). Notably, grid membranes of Structure 1 continue to manifest anisotropic wettability under varying strain conditions.

The wettability of intricate structures is fundamentally dictated by the synergistic interplay between material wettability and microstructural features. As indicated by Figure 6a, in Structure 1, filaments that are parallel within layers N and N + 1 generate surface tensions that are directionally symmetrical and equal in magnitude. However, the variation in Laplace forces—attributable to the droplets’ differing curvatures in these adjacent layers—prompts the droplets to extend along the X-axis. In contrast, Structure 2 exhibits uniform surface tensions in both the X and Y directions within a single layer, consequently exhibiting a consistent CA. Figure 8a illustrates that an increase in tensile strain prompts the three-phase contact line to migrate along the filament’s contour, altering the CA from *θ_A_* to *θ_A_*′. Notably, the intrinsic CA of the material (*θ_B_*), remains unaffected by strain variations.

### 3.5. Oil–Water Separation Properties of the PTFE/PDMS Grid Membrane

To characterize the oil–water separation properties, PTFE/PDMS grid membranes were positioned between two glass tubes, assembling a custom-made filtration unit. Initially, the flux of the grid membrane was assessed using this filtration unit, ensuring a consistent liquid height of 100 mm in the top tube. Next, the liquid was incrementally injected into the top tube at a pace of 0.2 mL/min until filtration through the grid membrane became unstable. The intrusion pressure (*P_int_*) is defined as the maximum hydrostatic pressure the grid membrane can withstand before the liquid in the top tube becomes unstable and descends into the lower tube. When the liquid transitions from the upper tube, passing through the grid membrane and reestablishing stability, the hydrostatic pressure it exerts on the grid membrane is labeled the stable intrusion pressure (*P_sta_*). At last, a mixture of oil (chloroform, CHCl_3_ dyed with Oil Red O) and water (deionized water dyed with methylene blue), in a 1:1 volume ratio totaling 10 mL, was simultaneously injected into the top tube. Owing to the hydrophobic and oleophilic properties of the grid membrane, CHCl_3_ passages through the PTFE/PDMS membrane, while water is retained within the upper tube. The volume of water remaining in the top tube was gathered and quantified using a volumetric cylinder. The efficiency of oil–water separation *E_sep_* was determined using the following equation:

*E_sep_* = *V_r_*/*V* × 100%, (3)

where *V_r_* is the volume of the rejected water in the upper tube, and *V* is the initial volume of water.

**Figure 9:**
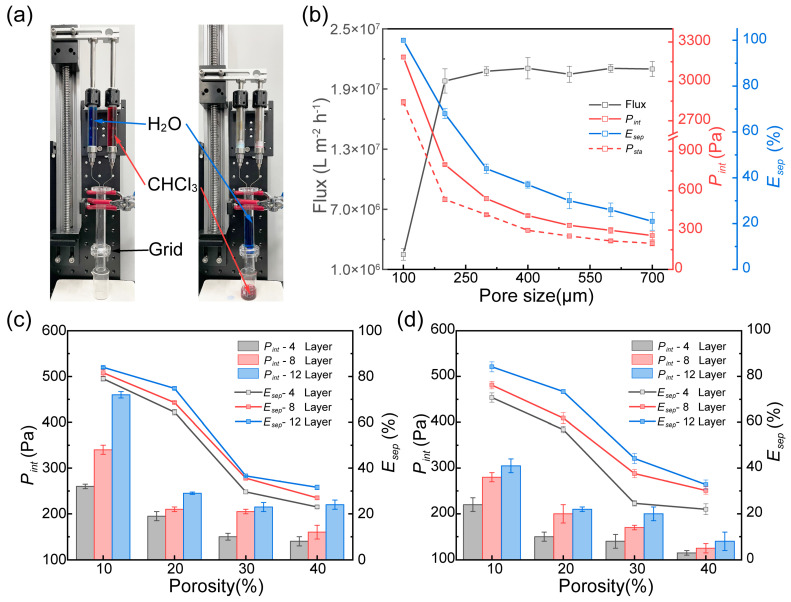
Oil–water separation properties of the PTFE/PDMS grid membrane. (**a**) Oil–water separation units and process. (**b**) Separation properties of single-pore membranes. Separation properties of grid membranes of (**c**) Structure 1 and (**d**) Structure 2.

Figure 9 illustrates the oil–water separation performance of DIW 3D-printed PTFE/PDMS grid membranes. With a pore size of 100 μm, the water flux of the sample is relatively low (2.4 × 10^6^ L·m^−2^·h^−1^), but the oil–water separation efficiency reaches 100%. This high efficiency is attributable to the high intrusion pressure, which effectively prevents water from infiltrating the pores, even when the stability of the water phase is compromised. As pore dimensions increase, water flux rises; however, this is accompanied by a significant degradation in oil–water separation efficiency. The failure of oil–water separation arises not only from the grid membranes’ insufficient intrusion pressure but also due to oil flow destabilizing the meniscus. For instance, a sample of 200 μm pore size provides an intrusion pressure of 798.1 Pa and a stable intrusion pressure of 532.8 Pa. Calculations indicate that under these conditions, the upper tube has a capacity of 7.7 mL; however, only 3.4 mL of water ultimately remains. Consequently, it is manifest that an increase in grid pore size not only diminishes intrusion pressure but also destabilizes the meniscus beneath the membrane, thereby undermining oil–water separation efficacy.

Figure 9c,d illustrate the oil–water separation performance of DIW 3D-printed grid membranes with different infill patterns. It can be observed that the intrusion pressure of the grid membrane is consistent with that of a single-pore membrane; furthermore, variations in the infill pattern do not affect intrusion pressure, while an increase in the number of layers enhances the membrane’s intrusion pressure. This can be attributed to the fact that, as a quasi-static parameter, intrusion pressure is intrinsically linked to the stability of the meniscus at the grid membrane’s bottom surface. The Laplace resultant force, inherent to a pendant droplet, arises from surface tension, and its magnitude is not only correlated with pore size but also dictates the level of intrusion pressure. Therefore, it can be observed that as porosity increases, the intrusion pressure significantly decreases. Regarding the separation efficiency, an increase in layer thickness has been shown to be effective. This improvement is because extended pore lengths minimize phase interfacial disturbances during the oil–water separation process. Similarly, as porosity increases, the separation efficiency is also substantially reduced. Thus, it can be concluded that intrusion pressure is an essential factor in determining the efficiency of oil–water separation, and the intrusion pressure is directly influenced by porosity. For grid membranes of Structure 1, liquid can passage not only vertically through the pores but also laterally, forming interconnected fluid pathways (Figure S8, Supporting Information). Such an arrangement further mitigates disturbances to the meniscus underneath the grid membrane during the injection of the oil–water mixture.

## 4. Conclusions

In this study, DIW 3D-printing technology was employed to fabricate PTFE/PDMS grid membranes. These grid membranes exhibited anisotropic hydrophobic characteristics, facilitating efficient oil–water separation. PTFE acted as a thixotropic agent and hydrophobic filler in the creation of PDMS-based composite inks. Rheological analyses of composite inks with varying proportions revealed that 60% PTFE/PDMS ink, which exhibits shear thinning and viscoelastic inversion properties, is suitable for DIW 3D printing. Precision in grid membrane manufacturing was attained through systematic investigation of the interrelationships among extruded filament stability, single filament dimensions, 3D-printed grid membrane features, and printing parameters. Optimization of factors such as extrusion air pressure, layer thickness, and printing speed enabled the printing of grids with diverse pore sizes. Three distinct printing states—printable, accumulated, and discontinuous—were identified under varying air pressure and printing speed conditions. These states informed our understanding of how extrusion air pressure and printing speed affect filament width. By manipulating the pore size, grids exhibiting superhydrophobic characteristics were fabricated, and their CAs varied anisotropically in the parallel and vertical directions. Subsequent studies explored the wettability of grid membranes with different pore sizes, delving into the relationships among droplet volume, grid membrane strain, and contact angle in Cassie–Baxter contact states without a bottom grid. Finally, the grid membranes’ utility in oil–water separation was confirmed, with certain configurations achieving a 100% separation efficiency. This study found that the separation performance depended on factors like the intrusion pressure, grid structure, and number of layers. This research substantiates the potential of DIW 3D-printing technology in the design and optimization of surfaces with specialized wettability features, such as anisotropy and superhydrophobicity.

## Figures and Tables

**Table 1:** The mechanical strengths of the PTFE/PDMS samples.

PTFE Content/wt%	Tensile Strength (MPa)	Elongation at Break (%)
0	3.85 ± 0.31	82.16 ± 5.15
50	4.03 ± 0.22	132.11 ± 8.12
55	2.44 ± 0.18	101.27 ± 6.31
60	1.81 ± 0.10	42.42 ± 6.88
65	0.78 ± 0.13	11.69 ± 4.45

## Data Availability

All data generated or analyzed during this study are included in this published article.

## Supplementary Materials

The following supporting information can be downloaded at https://www.mdpi.com/article/10.3390/polym17020174/s1, Figure S1: Photos of DIW 3D-printed PTFE/PDMS grid membranes; Figure S2: (a) Image of the custom-made DIW 3D printer for printing the PTFE/PDMS composite ink, and (b) enlarged image showing the DIW 3D-printed grid membrane; Figure S3: Measurement of the length of filament extruded onto a water surface; Figure S4: Regulation of filament spacing (a) LT = 0.15 mm and (b) LT = 0.35 mm; Figure S5: Photos of DIW 3D-printed PTFE/PDMS filaments; Figure S6: CA of grid membrane of Structure 1 in the (a) X direction and (b) Y direction; Figure S7: Photos of CA measurements for the PTFE/PDMS grid membranes of (a) Structure 1 and (b) Structure 2 under strain conditions; Figure S8: Photos of separation properties measurements for grid membranes. (a) Bottom-view images during intrusion pressure measurement. (b) Side-view images illustrating liquid flow through a cavity.
