# Supplementary material for: Direct Ink Writing 3D Printing Polytetrafluoroethylene/Polydimethylsiloxane Membrane with Anisotropic Surface Wettability and Its Application in Oil–Water Separation
Source: Polymers (Basel). 2025 Jan 13;17(2):174. doi: 10.3390/polym17020174 (PMC11768408; doi:10.3390/polym17020174)
Supplement: Supplementary file 1 [file polymers-17-00174-s001.zip › polymers-3396156-supplementary.pdf]

Direct Ink Writing 3D Printing PTFE/PDMS Membrane With Anisotropic Surface Wettability and Its Application in Oil–water Separation

Geng Peng [1,*] and Jiang Chengjian [1]

[1] State Key Laboratory of Material Processing and Die & Mold Technology, School of Materials Science and Engineering, Huazhong University of Science and Technology, Wuhan 430074, China; gengpeng@hust.edu.cn; jiang_cj@hust.edu.cn

* Correspondence: gengpeng@hust.edu.cn

Supplementary material

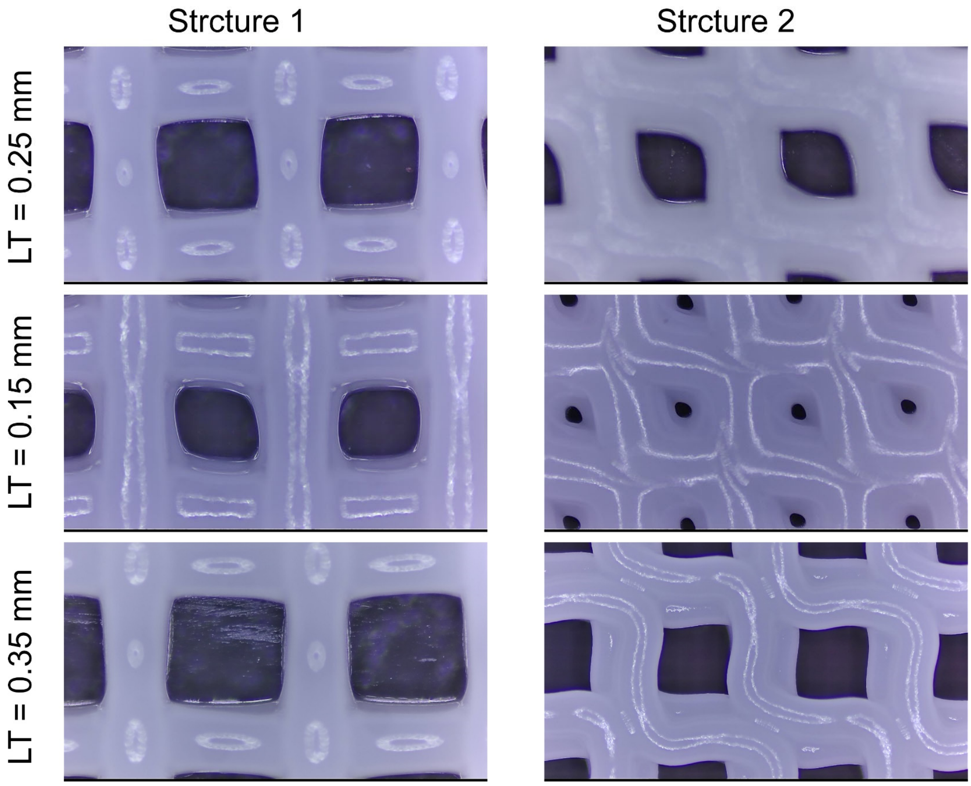

Figure S1 Photos of DIW 3D-printed PTFE/PDMS grid membranes

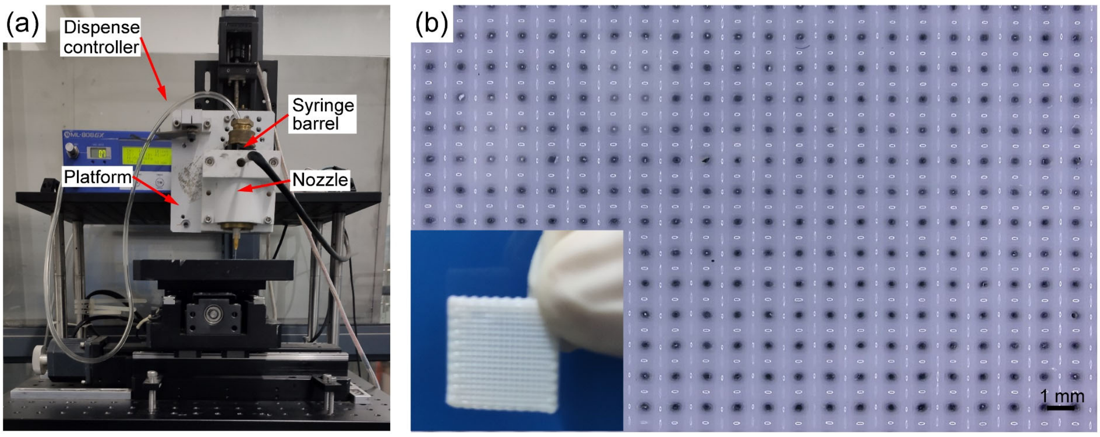

Figure S2 (a) Image of the custom-made DIW 3D printer for printing the PTFE/PDMS composite ink, and (b) enlarged image showing the DIW 3D-printed grid membrane.

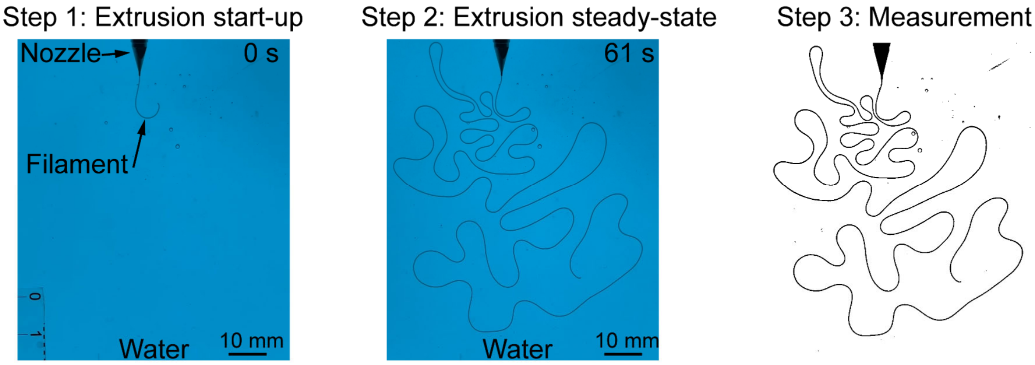

Figure S3 Measurement of the length of filament extruded onto a water surface.

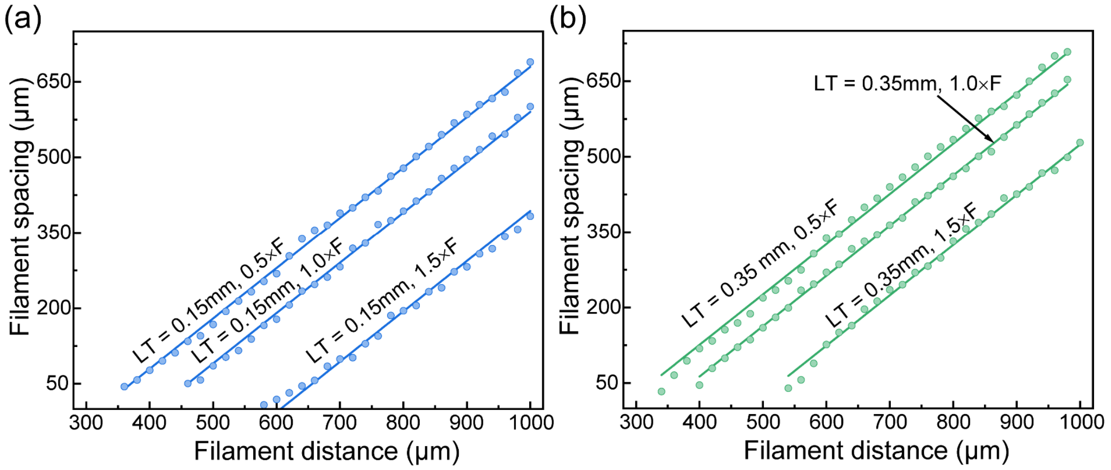

Figure S4 Regulation of filament spacing (a) *LT* = 0.15 mm and (b) *LT* = 0.35 mm.

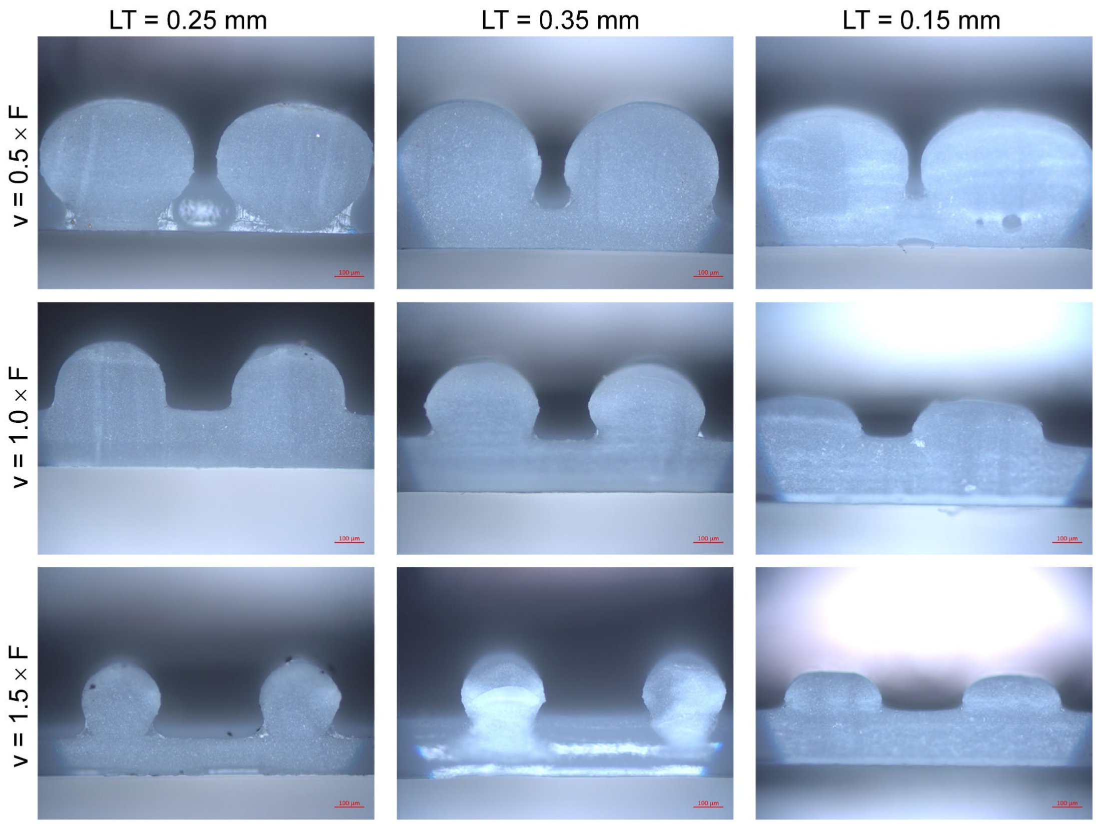

Figure S5 Photos of DIW 3D-printed PTFE/PDMS filaments

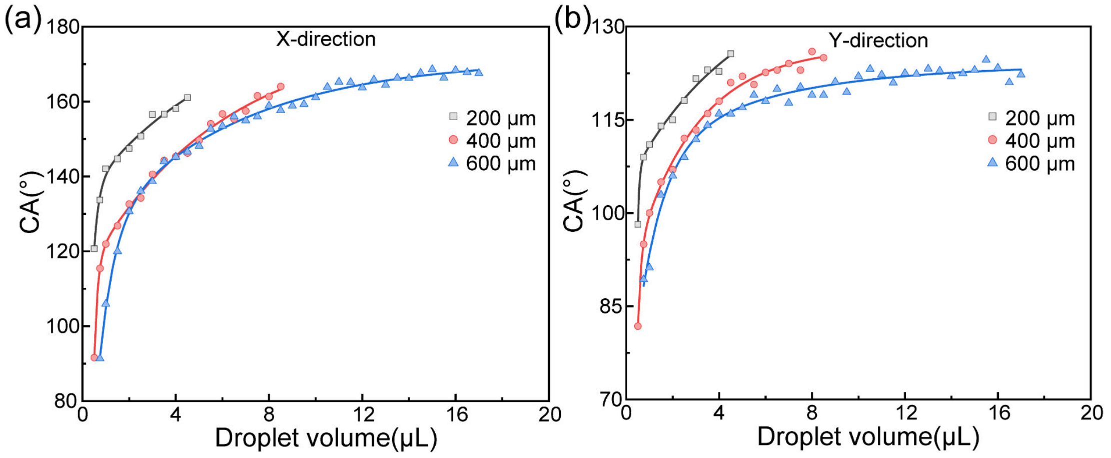

Figure S6 CA of grid membrane of Structure 1 in the (a) X direction and (b) Y direction.

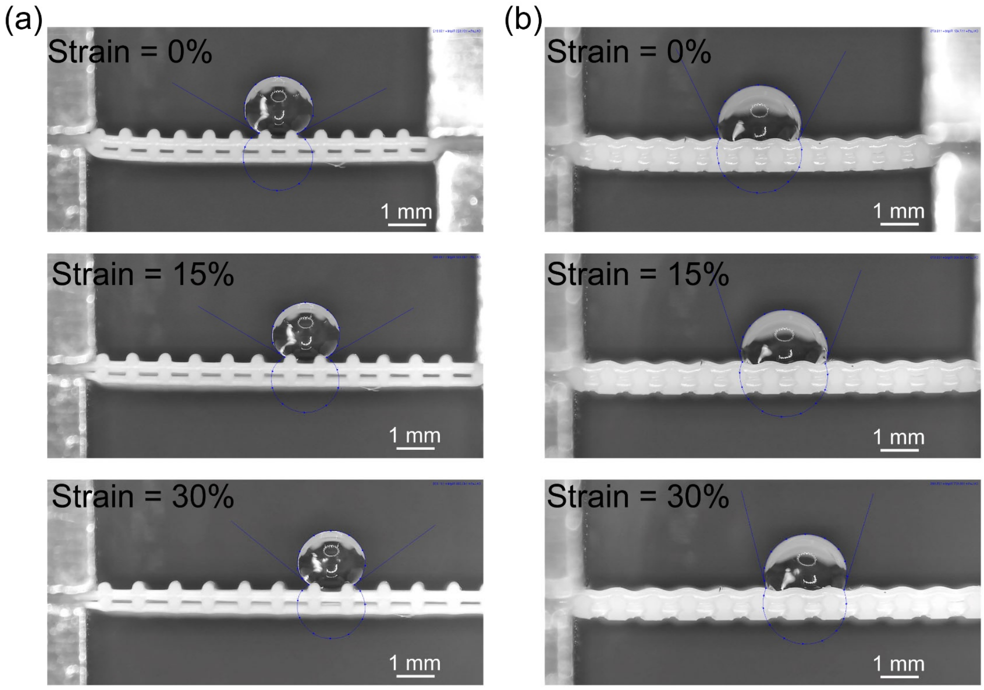

Figure S7 Photos of CA measurements for the PTFE/PDMS grid membranes of (a) Structure 1 and (b) Structure 2 under strain conditions.

(a)

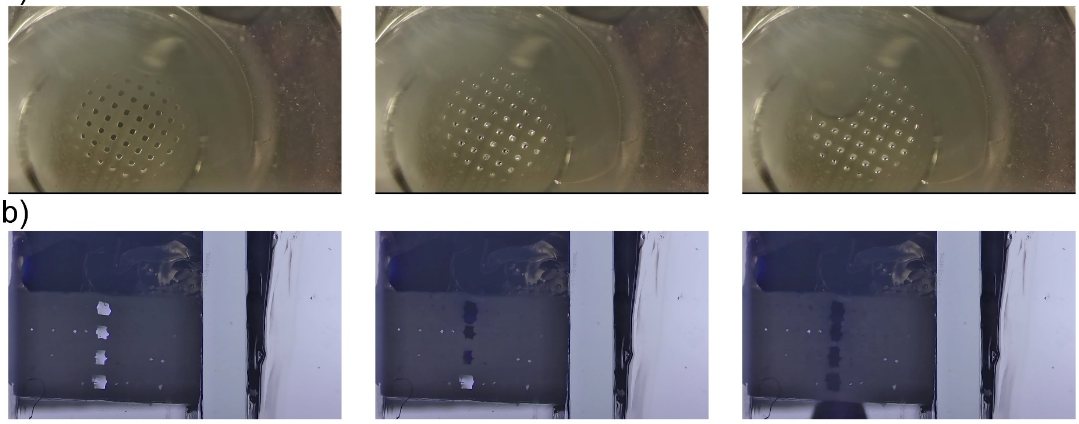

(b)

Figure S8 Photos of separation properties measurements for grid membranes. (a) Bottom-view images during intrusion pressure measurement. (b) Side-view images illustrating liquid flow through a cavity.
